# Arterial Blood Pressure Features of Hypertensive Patients with Typical and Atypical 460 nm Skin Fluorescence Response to Transient Ischaemia

**DOI:** 10.3390/jcm12185886

**Published:** 2023-09-10

**Authors:** Regina Pawlak-Chomicka, Paweł Uruski, Tomasz Krauze, Jarosław Piskorski, Andrzej Tykarski, Przemysław Guzik

**Affiliations:** 1 Department of Hypertensiology, Angiology and Internal Medicine, Poznan University of Medical Sciences, 61-848 Poznan, Poland; pawlakregina@gmail.com (R.P.-C.); puruski@ump.edu.pl (P.U.); tykarski@ump.edu.pl (A.T.); 2 Department of Cardiology-Intensive Therapy and Internal Medicine, Poznan University of Medical Sciences, 60-355 Poznan, Poland; tomaszkrauze@wp.pl; 3 Institute of Physics, University of Zielona Gora, 65-516 Zielona Gora, Poland; jaropis@zg.home.pl

**Keywords:** arterial hypertension, flow mediated skin fluorescence (FMSF), ischaemia, pulse wave

## Abstract

Flow-mediated skin fluorescence (FMSF) at 460 nm is a non-invasive method for assessing dynamic changes in the reduced form of nicotinamide adenine dinucleotide (NADH) and microcirculation in forearm skin under varying conditions of tissue perfusion. Typically, fluorescence increases during ischaemia, but atypical cases show a temporary signal decrease instead of a constant increase. This study aimed to explore the clinical implications of atypical FMSF patterns in patients with newly diagnosed untreated hypertension. NADH fluorescence and pulse wave analysis were performed on 65 patients. Differences in peripheral and arterial pulse pressure profiles were examined based on FMSF curve courses. Patients with atypical curve courses had significantly (*p* < 0.05 or lower for all) higher heart rate, peripheral and central diastolic pressure, tension time index, central rate pressure product, shorter diastole duration, and reservoir pressure–time integral. Hypertensive patients with atypical FMSF signals had less advantageous blood pressure profiles. Although the underlying factors causing these symptoms are unknown, the atypical FMSF pattern may reflect increased sympathetic stimulation and vascular resistance. The visual assessment of the FMSF curve may have important clinical implications that deserve further investigation.

## 1. Introduction

The reduced form of nicotinamide adenine dinucleotide (NADH) is a particle synthesised in the mitochondria (most of it) and in the cellular cytoplasm. Under aerobic conditions, it is converted to an ionic form by participating in many metabolic pathways. NADH is essential for the production of ATP. NADH accumulates in the cytoplasm during hypoxia/anoxia due to its inefficient oxidation to NAD+ by mitochondria [1,2].

**Figure 1:**
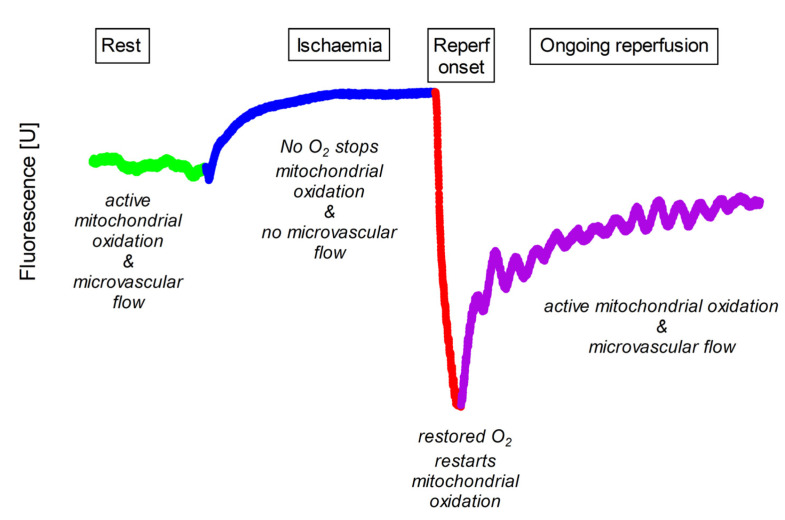
Typical pattern of 460 nm skin fluorescence acquired at rest (Rest), during transient ischaemia (Ischaemia) and during initial (Reperf onset) and late (Ongoing reperfusion) phases of reperfusion. Rest is shown in light green, ischaemia in blue, initial reperfusion in red and late reperfusion in purple. Compared to the resting state, the fluorescence signal gradually increases during ischaemia. After the onset of reperfusion there is a rapid decrease to a local nadir, and during late reperfusion the fluorescence signal gradually recovers to values close to the pre-ischaemic rest.

Flow-mediated skin fluorescence (FMSF) at 460 nm is a non-invasive method for assessing dynamic changes in NADH and microcirculation in response to transient global ischaemia and subsequent reperfusion. FMSF measures the NADH fluorescence signal in the skin of the forearm in three phases: rest, ischaemia and post-ischaemic reperfusion (Figure 1) [1,2].

First, FMSF detects the fluorescence signal at rest with normal tissue perfusion. Blood flow is always present during the resting phase. Therefore, skin fluorescence reflects the influence of both cellular metabolism and microcirculatory effects on NADH concentration in superficial keratinocytes.

Next, skin fluorescence is measured during ischaemia with total, transient arterial occlusion and no blood flow. Transient total ischaemia is then induced by completely occluding the brachial artery with a blood pressure cuff inflated to 60 mmHg above systolic pressure [3]. When blood flow is blocked, the ischaemic phase provides a unique window to observe cellular metabolism as aerobic processes are gradually extinguished and anaerobic pathways, including cytoplasmic glycolysis, the source of NADH, gain an advantage.

Finally, skin fluorescence is measured during reperfusion when the brachial cuff is deflated and blood flow to the skin is restored. During reperfusion, skin NADH levels are influenced by both cellular metabolism and the returning microcirculation. Initially, reperfusion is characterised by a sharp drop in the NADH signal. The supply of oxygen to the mitochondria allows them to immediately oxidise the previously accumulated reduced form to NAD+. The fluorescence signal then reaches its lowest value. After a rapid decrease, a slow return to the basal state is observed, with numerous oscillations of the signal related to the blood flow [1].

The FMSF method has been used to investigate healthy people and athletes at rest and during various activities [4]. It has also been applied to patients with coronary artery disease [5], diabetes [6], obstructive pulmonary disease [7] and lupus [8].

Resting cellular NADH increases with age and in several cardiovascular diseases [2,6], obstructive pulmonary disease [7] and diabetes [6]. Patients with arterial hypertension (HTN) differ from healthy subjects in the early phase of the FMSF curve during limb ischaemia because they accumulate NADH more rapidly [9]. Further, we have recently shown that metoprolol increases NADH fluorescence in patients with HTN, an effect not seen with amlodipine, perindopril or nebivolol [10].

Typically, 460 nm fluorescence increases during ischaemia. However, atypical responses are possible. Sometimes, there is no visible increase in the 460 nm skin fluorescence. The fluorescence signal decreases or fluctuates up and down during ischaemia.

Such atypical patterns were initially thought to reflect the movement of the forearm when being tested [11]. However, restricting the movements of the patient did not correct the abnormalities. In addition, some individuals recorded atypical patterns, suggesting that it is a subject-dependent feature. As the exact mechanisms are unclear, possible explanations include changes in arterial blood flow, microvascular properties, endothelial dysfunction or altered mitochondrial function. All of these phenomena are found in patients with HTN [9].

No study has examined the clinical features of patients with HTN with typical and atypical FMSF [11]. This study aimed to compare the clinical characteristics of newly diagnosed untreated HTN patients with typical and atypical FMSF.

## 2. Materials and Methods

### 2.1. Study Group

Sixty-five patients with newly diagnosed primary HTN were enrolled in the study. Primary HTN was diagnosed based on 24 h ambulatory blood pressure monitoring (ABPM) according to the European Society of Hypertension 2018 guidelines [12]. Two clinical inclusion criteria were used for patient enrolment: (1) newly diagnosed and previously untreated primary HTN with systolic blood pressure (SBP) <180 and/or diastolic blood pressure (DBP) <110 mmHg, and (2) sinus rhythm electrocardiogram. The exclusion criteria included secondary HTN, other chronic diseases such as diabetes, chronic obstructive pulmonary disease, cancer, congestive heart failure, atrial fibrillation, renal impairment (with estimated glomerular filtration rate <60 mL/min/1.73 m^2^), acute inflammation, pregnancy or lactation.

### 2.2. Ambulatory Blood Pressure Monitoring (ABPM)

ABPM was performed on all patients using the AND-TM 2430 monitor (A&D Company, Limited; Tokyo, Japan). ABPM was used for blood pressure assessment because of the accuracy of the measurement [13,14]. The measurement was made on the limb with higher pressure when the difference was significant, or on the left arm if there was no significant difference.

Following the ESH 2018 guidelines, each ABPM device measured blood pressure every 15 min between 6 am and 10 pm and every 30 min between 10 pm and 6 am. Day and night times were determined individually according to the hours reported by the patient. ABPM values are on average lower than office BP values, and the diagnostic threshold for HTN is ≥130/80 mmHg over 24 h, ≥135/85 mmHg for the daytime average or ≥120/70 mmHg for the nighttime average. All these values are equivalent to office BP ≥140/90 mmHg.

The parameters describing the ABPM, calculated as the average of the collected measurements, are listed below:

- Heart rate [beats/min] for daytime (DTHR) and nighttime (NTHR);
- Systolic blood pressure [mmHg] for the day (DTSBP) and night (NTSBP);
- Diastolic blood pressure [mmHg] for the day (DTDBP) and night (NTDBP).

The diagnostic threshold for HTN, based on the ABPM, was ≥130/80 mmHg over 24 h or ≥135/85 mmHg for the wake time average, or ≥120/70 for the sleep time.

### 2.3. Flow-Mediated Skin Florescence (FMSF) and Division of the Study Group

The skin emits 460 nm fluorescent light in response to excitation by 340 nm ultraviolet light. As the UV light does not penetrate deeper than 0.5 mm from the skin surface, the FMSF method provides information from the most superficial skin cells [5,15]. However, this model gives a general picture of the state of the body’s microcirculation [16].

**Figure 2:**
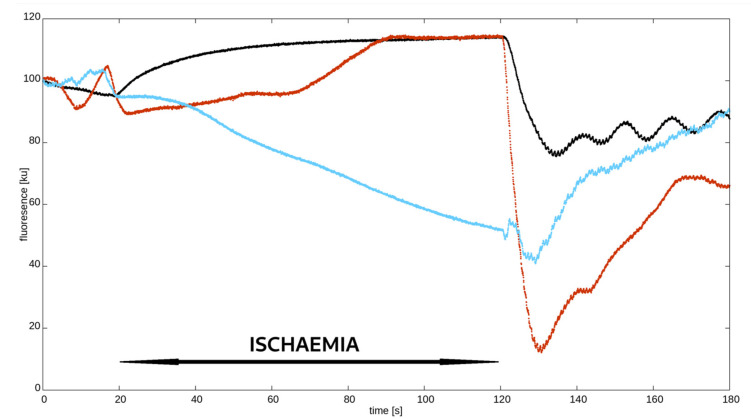
Synchronised examples of different nicotinamide adenine dinucleotide (NADH) fluorescence curves measured using flow-mediated skin fluorescence (FMSF). Black—typical pattern; red and blue—atypical pattern.

As described in previous studies on FMSF, skin fluorescence at 460 nm typically increases and reaches a plateau in response to ischaemia. During reperfusion, this fluorescence rapidly decreases to a minimum value, which is always below the baseline, and then gradually increases to pre-ischaemic values. As mentioned above and illustrated in Figure 2, this pattern is missing in some people, and the NADH curve during ischaemia has an atypical shape. Based on a visual inspection, i.e., the eyeballing, of the ischaemic NADH pattern, we divided hypertensive patients into two groups:

- Typical response—those with an expected increase and plateau in 460 nm skin fluorescence during ischaemia;
- Atypical response—the remaining patients with a decrease, no increase, delayed and short-lived, or multimodal with brief increases and decreases in fluorescence.

Due to the unpredictable and non-uniform nature of atypical ischaemic patterns, no single mathematical parameter derived from linear regression analysis was able to distinguish atypical from typical patterns. Therefore, we decided to use the eyeballing approach, which seems to be the simplest and quickest method to differentiate between typical and atypical patterns.

### 2.4. Non-Invasive Assessment of the Radial and Central Arterial Pressure Waveforms

Radial pressure waveforms were recorded non-invasively using a piezoelectric tonometer (Colin BPM 7000, Colin, Sue, Japan). The recorded analogue signal was processed in real time using SphygmoCor software Mx, (Version 7.0, AtCor Medical, Sydney, NSW, Australia) for online reconstruction (using a validated transfer function) of a pressure waveform characteristic of an ascending aorta. Pulse wave analysis was used to assess peripheral and central haemodynamics. Both pressure waveforms were averaged using the Sphygmocor software (Version 7.0) and the measured and derived parameters are shown in Table 1.

The reservoir–excess pressure model decomposes the arterial blood pressure waveform into three components: reservoir pressure, excess pressure and diastolic blood pressure (DBP). The reservoir pressure is the dominant component of the measured diastolic pressure, while the excess pressure is largely responsible for the early systolic pressure rise [17]. The reservoir pressure reflects the compliance of the arterial system and the ventricular–arterial coupling, while the excess pressure reflects wave reflection phenomena, vascular resistance and left ventricular afterload and ejection [18]. On the other hand, DBP reflects the minimum pressure in the arteries and is important for maintaining minimum blood flow and perfusion to the body’s organs and tissues.

The time integral of the whole arterial pressure waveform is the total blood pressure time integral (TBTI) and has three separate components: the reservoir pressure time integral (RPTI), excess pressure time integral (EPTI) and diastolic pressure time integral (DBPTI) [16]. The RPTI represents the work performed by the heart to fill and keep the arterial system distended. In contrast, the EPTI represents the work performed by the heart to overcome wave reflections and peripheral resistance, and to distend its lumen above the diastolic value [17]. Therefore, the RPTI and EPTI can provide physiological and clinical insights into cardiovascular function and disease [17,18].

The following parameters were derived from the pulse wave analysis (Table 1). For the excess and reservoir pressure indices, we used in-house software developed in Python (Version 3.8, Python Foundation, Ipswich, MA, USA) according to the published methodology [19,20,21,22,23,24,25,26].

### 2.5. Ethics Approval Statement

The study was designed and conducted following the tenets of the Declaration of Helsinki. The Bioethics Committee of the Medical University of Poznan approved the study (approval no. 435/17, Appendix 243/19). Written informed consent was obtained from all participants.

### 2.6. Statistical Analysis

Graphical analysis of data distribution (histograms and Q-Q plots) and the Shapiro–Wilk test showed that the distribution of continuous data was not normal; therefore, all data are summarised as median and 25th and 75th percentiles (Q1–Q3). The non-parametric Mann–Whitney test was used to compare patients with typical and atypical FMSF curves. Differences with a *p*-value < 0.05 were considered statistically significant. All statistical analyses were performed with JMP Pro 17.

## 3. Results

Women accounted for 38% of all patients studied. Atypical ischaemic patterns were observed in 32.3% of patients. In the groups with typical and atypical FMSF curves, women accounted for 32% and 52%, respectively. No differences were found in the median age (38.5 (30.8–49.0) vs. 43 (37.0–48.5) years old; *p* = 0.32) and body mass index (27.7 (24.5–31.8) vs. 28.7 (25.3–34.1) kg/m^2^; *p* = 0.53) between these groups. The comparison of various features of blood pressure and radial and central pulse wave analysis are shown in Table 2. The youngest and oldest patients recruited were 19 and 66 years of age, respectively.

The groups did not differ in blood pressure as measured with the ABPM. However, there was a statistically significant difference in the mean nighttime heart rate and some parameters describing radial and central pulse wave analysis.

Patients with an atypical skin fluorescence response to ischaemia had a faster resting heart rate and shorter diastolic duration, a reduced relative contribution of the diastole duration to the whole cycle length and reservoir pulse pressure integral of the central pulse wave. They also had significantly higher diastolic pressure, mean blood pressure, ratio of the time of arrival of the reflected wave to the ejection time in the peripheral artery and central parameters such as diastolic pressure, mean pressure during diastole, tension time index, diastolic time index and rate–pressure product.

## 4. Discussion

Patients with HTN and an atypical pattern of 460 nm skin fluorescence in response to transient ischaemia displayed significantly different pressure waveform characteristics compared to those with a typical FMSF shape. These patients had a faster resting and sleep heart rate, shorter absolute and relative diastolic duration, higher peripheral and central DBP, increased TTI and RPP and worse RPTI.

FMSF records 460 nm skin fluorescence at rest, during ischaemia and reperfusion. During the resting and reperfusion phases, the level of NADH fluorescence is significantly influenced by blood flow, particularly in the skin vascular bed. In contrast, no blood flow occurs during the total brachial artery occlusion and induced forearm ischaemia. The results provide a unique opportunity to gain insights into the primary potential of 460 nm fluorescence and its linear dependence on the NADH content.

Both mitochondria and cytoplasm contribute to NADH accumulation during ischaemia. Long hypoxia or anoxia reduces or ceases the oxidation of mitochondrial NADH, shifting anaerobic cytoplasmic glycolysis as a major source of cellular energy [27]. However, determining which of these two mechanisms makes a greater relative contribution to NADH accumulation during brief skin ischaemia remains uncertain.

The cytoplasmic NAD/NADH ratio can be much higher than the mitochondrial NAD/NADH ratio in a typical eukaryotic cell. Therefore, cytoplasmic glycolysis may have a greater impact on NADH accumulation during ischaemia. However, this may not be true for all cell types, as some have a higher mitochondrial density and lower glycolytic capacity. In cardiomyocytes, the mitochondrial origin of NADH may be more important for its accumulation during ischaemia. However, NADH metabolism in the skin cells may be different from cardiomyocytes [28].

Keratinocytes, the predominant cell type in the epidermis, have a low mitochondrial density and a distinct glycolytic phenotype compared to other cell types. NADH accumulation during skin ischaemia in the keratinocytes depends on the balance between glycolysis and mitochondrial respiration. Keratinocytes possess fewer mitochondria, exhibiting a discernible reduction in cristae abundance with limited oxidative phosphorylation potential [29]. If glycolysis is more active than respiration, NADH may accumulate in the cytoplasm due to the production of lactate during ischaemia. If respiration is more active than glycolysis, NADH may accumulate in the mitochondria due to the inhibition of complex I of the electron transport chain by induced hypoxia/anoxia. Therefore, both mechanisms may contribute to the keratinocytes’ NADH accumulation during skin ischaemia.

With ageing, numerous mtDNA mutations accumulate in the mitochondria of the skin, accompanied by a decrease in mitochondrial function, an increase in the production of reactive oxygen species, and an increased mitophagy and apoptosis [30]. Hypertension is accompanied by inflammation, oxidative stress and endothelial dysfunction, which are also mechanisms of the body’s ageing process [31].

In recent years, evidence of the role of mitochondria in HTN pathogenesis has increased. Chronically elevated blood pressure damages mitochondria, causing their dysfunction, increasing active oxygen species production and disturbing cellular homeostasis [32,33]. The unexpected course of the FMSF curve during ischaemia, significantly different from the typical appearance, is probably the result of impaired metabolic processes associated with the loss of mitochondrial functionality. However, some adaptive changes in the cytoplasm of various cells, including keratinocytes, are also possible.

Patients with HTN may have typical and atypical ischaemia-related NADH accumulation regardless of the potential mechanisms. Individuals with an atypical ischaemic phase of the FMSF curve have significantly higher DBP-related indices, rate pressure product and cTTI compared to other patients with HTN. They also present with faster pulse rates, shorter duration of the diastole and worse RPTI.

The pulse rate and blood pressure elevation indirectly imply an increased sympathetic drive, afterload for the heart and myocardial oxygen consumption [34,35,36]. Sympathetic activation causes an increase in systemic vascular resistance, mainly by muscle arteries with a diameter of 100–450 um and pre-capillary arterioles [37]. Chronically elevated blood pressure causes harmful remodelling of the arterial wall, which can impair the transfer of oxygen and nutrients from the blood to the tissues. HTN causes changes in the composition and organisation of extracellular matrix proteins, including collagen and elastin. It also affects the interaction between the endothelium and the underlying smooth muscle cells. The passage of nutrients from the vessel lumen to the surrounding tissues and cells may be further impeded by damaged endothelium and thickened walls of the arterial tree.

The skin is at the end of the blood supply, so any process that impedes the transfer of oxygen and nutrients in the long term may lead to adaptive changes in the skin. Hypertension-induced changes to arterial walls affect the blood flow and oxygen delivery to tissues, leading to local inflammation and oxidative stress, hypertrophy, hyperplasia, apoptosis or fibrosis of the arterial wall cells. Larger arteries become stiffer, thicker and narrower, while hypertrophy of smooth muscle in smaller arteries increases vascular resistance [38]. Vascular resistance depends on several factors, including blood viscosity, vessel length and diameter, and compliance [39]. Vascular resistance is referred to as left ventricular (LV) afterload (the pressure that the LV must generate to open the aortic valve and pump blood into the aorta). During ejection, the LV must overcome the vascular resistance of the systemic circulation. Another contributor to LV afterload is arterial stiffness, which often increases in patients with HTN [40]. Vascular resistance affects the mean arterial pressure and, if elevated, can affect the perfusion pressure of organs and tissues. DBP contributes more to mean blood pressure than SBP and is strongly related to vascular resistance [41]. This may explain why patients with HTN with an atypical ischaemic phase of FMSF have abnormal values of several diastolic-related parameters.

EPTI is also influenced by vascular resistance, particularly in its early systolic pressure rise. Although indirect, vascular resistance contributes to RPTI as well. Reservoir pressure is determined by the compliance of the arterial system, which depends on the diameter and tone of the arteries. Both are modulated by vascular resistance. Further, a higher heart rate shortens the duration of diastole [42], reduces RPTI and worsens the blood supply to the coronary arteries perfused predominantly during diastole.

cRPP is a product of heart rate and central systolic pressure that indirectly reflects myocardial oxygen consumption. A lower rate–pressure product is beneficial and occurs in physically active people [34]. Myocardial oxygen consumption increases with afterload. From the results of our study, cTTI could be considered as a proxy for LV afterload. cTTI is higher in people with an atypical ischaemic phase of the FMSF curve. Gedikli et al. [43] showed that cTTI is higher in people diagnosed with hypertension than in healthy controls. High cTTI is typical in elderly people with stiffer arteries [44]. Higher cTTI in patients with atypical FMSF may indicate that their arterial walls are stiffer or that hypertension has caused some age-related adaptation of the arteries. Altogether, HTN presents a longer duration or is more advanced in patients with an atypical ischaemic phase of FMSF. Another possibility is that these patients are more sensitive to increased blood pressure and develop some features that suggest either a longer duration or more advanced HTN.

### 4.1. Study Limitations

We used a cross-sectional study design, which makes it impossible to assess causality. Only patients with untreated HTN were included. The FMSF measurement was performed only once for a given patient. The studied patients were adults aged between 19 and 66 years. Therefore, our results cannot be extrapolated to children and adults over 66 years of age. Only indirect parameters characterising arterial stiffness, afterload and vascular resistance were used. However, these parameters were measured non-invasively. In addition, this is a post hoc analysis of an already completed study that cannot be extended [9]. To identify typical and atypical ischaemic phases of the FMSF curve, we used visual inspection, which may be another study limitation. The analysis of any curve or signal should start with the identification of its shape by eye. Later, if possible, the curve can be quantified using mathematical descriptors. Our experience shows that for the typical ischaemic response, the pattern can be generalised into one. For atypical patterns, however, there are many possibilities and no single mathematical parameter that we have tested has proved effective. Furthermore, eyeballing is very often a common practice, even for diagnostic purposes such as electrocardiography. The shapes of P, T and U waves, QRS complexes and their interrelationships and different patterns were studied to make a diagnosis. Next, this study compared several parameters characterising blood pressure and pulse wave between patients with typical and atypical FMSF curves. We know that multiple comparisons may produce some random significant differences by chance. Therefore, our study should be considered exploratory. Finally, this study did not investigate the effects of HTN treatment on the shape of FMSF and the presence or absence of the typical ischaemic phase of FMSF. It is unclear whether any treatment could reverse an atypical FMSF pattern.

### 4.2. Perspectives

The atypical pattern of the FMSF curve in patients with HTN may help to identify those with more advanced disease, longer duration or greater sensitivity to its clinical consequences. As such patients appear to have a more activated sympathetic nervous system, they may benefit from treatment with antiadrenergic drugs. Whether profiling of patients with HTN with FMSF as those with a typical and atypical ischaemic response could help to personalise antihypertensive therapy requires further study.

It might be a good idea to develop an automated tool for analysing the FMSF signal, which would quantify the results and detect whether the ischaemic phase is typical or not. However, such a tool would require modelling with more training data and validation of the obtained models and parameters with an independent data set.

## 5. Conclusions

Patients with HTN and atypical FMSF have a less favourable blood pressure profile. The atypical course of the NADH fluorescence curve in hypertensive patients indicates a different phenotype of the disease that may be secondary to increased sympathetic stimulation.

## Figures and Tables

**Table 1:** Parameters obtained from the analysis of the pulse wave.

Parameter	Explanation
HR	Heart rate [beats/min]
DD	Diastole duration [ms]—the duration of ventricular relaxation which reflects most of the coronary perfusion time
pSBP	Peripheral systolic blood pressure [mmHg]
pDBP	Peripheral diastolic blood pressure [mmHg]
cSBP	Central systolic blood pressure [mmHg]
cDBP	Central diastolic blood pressure [mmHg]
cTTI	Central tension time index—an index of the myocardial oxygen demand per beat. It is calculated by multiplying the central systolic blood pressure by the systolic time interval. It reflects the afterload, i.e., the effects of blood pressure during left ventricle systole.
cRPP	Central rate pressure product [mmHg x beats/min], cRPP = cSBPxHR. It is an indirect measure of myocardial oxygen consumption
EPTI	Excess pressure–time integral of central pulse wave—the area under the excess pressure curve
RPTI	Reservoir pressure–time integral of central pulse wave—the area under the reservoir pressure

**Table 2:** Median age, ambulatory blood pressure monitoring (ABPM) and radial and central pulse waves analysis in the patients with typical and atypical flow-mediated skin fluorescence (FMSF) pattern.

	Typical Pattern N = 44		Atypical Pattern N = 21		*p*-Value (Mann–Whitney Test)
	Median	Q1–Q3	Median	Q1–Q3	
DTSBP [mmHg]	148.4	142–156.5	147.2	143–156.4	0.84
DTDBP [mmHg]	87.6	83.1–92.3	91.6	84.9–95.9	0.09
DTHR [beats/min]	81.7	76–89.9	83.1	76.4–89.7	0.95
NTSBP [mmHg]	122.7	114.1–130.1	119	110.3–140.7	0.83
NTDBP [mmHg]	70	65.4–75.9	70.5	65.5–82.4	0.44
NTHR [beats/min]	63.2	57.7–71.2	68.6	63.6–76.9	0.03
HR [beats/min]	69.5	61.6–80.9	75.3	71.2–85	0.03
DD [ms]	547.4	445.5–647	506.5	427–532.7	0.02
pSBP [mmHg]	129.7	120.3–137.1	129.5	124.8–139.4	0.5
pDBP [mmHg]	78.8	74.7–85.1	86.6	78.8–92	0.03
cSBP [mmHg]	113.3	107.3–126.1	119.2	112.1–126.6	0.23
cDBP [mmHg]	80.5	75.9–86.6	87.2	79.7–93.3	0.03
cTTI [mmHg × s^−1^ × min^−1^]	2320.1	2118.1–2653.1	2524.7	2341.5–2764	0.02
cRPP [mmHg × beats/min]	7805.5	7111.9–9553.1	8801.2	8188.3–10,196.4	0.02
EPTI [mmHg × ms]	4.6	4.2–5.2	4.3	3.8–4.8	0.05
RPTI [mmHg × ms]	10.3	7.7–11.4	8	7–10.1	0.03

Abbreviations: DTSBP—daytime systolic blood pressure; DTDBP—daytime diastolic blood pressure; DTHR—daytime heart rate; NTSBP—nighttime systolic blood pressure; NTDBP—nighttime diastolic blood pressure; NTHR—nighttime heart rate; HR—heart rate; DD—diastole duration; pSBP—peripheral systolic blood pressure; pDBP—peripheral diastolic blood pressure; cSBP—central systolic blood pressure; cDBP—central diastolic blood pressure; cTTI—central tension time; cRPP—central rate pressure product; EPTI—excess pressure–time integral of central pulse wave; RPTI—reservoir pressure–time integral of central pulse wave.

## Data Availability

Not applicable.
